# A Genome-Wide Association Study of Circulating Galectin-3

**DOI:** 10.1371/journal.pone.0047385

**Published:** 2012-10-09

**Authors:** Rudolf A. de Boer, Niek Verweij, Dirk J. van Veldhuisen, Harm-Jan Westra, Stephan J. L. Bakker, Ron T. Gansevoort, Anneke C. Muller Kobold, Wiek H. van Gilst, Lude Franke, Irene Mateo Leach, Pim van der Harst

**Affiliations:** 1 Department of Cardiology, University of Groningen, University Medical Center Groningen, Groningen, The Netherlands; 2 Department of Genetics, University of Groningen, University Medical Center Groningen, Groningen, The Netherlands; 3 Department of Internal Medicine, University of Groningen, University Medical Center Groningen, Groningen, The Netherlands; 4 Department of Laboratory Medicine, University of Groningen, University Medical Center Groningen, Groningen, The Netherlands; 5 Durrer Center, Utrecht, The Netherlands; Kunming Institute of Zoology, Chinese Academy of Sciences, China

## Abstract

Galectin-3 is a lectin involved in fibrosis, inflammation and proliferation. Increased circulating levels of galectin-3 have been associated with various diseases, including cancer, immunological disorders, and cardiovascular disease. To enhance our knowledge on galectin-3 biology we performed the first genome-wide association study (GWAS) using the Illumina HumanCytoSNP-12 array imputed with the HapMap 2 CEU panel on plasma galectin-3 levels in 3,776 subjects and follow-up genotyping in an additional 3,516 subjects. We identified 2 genome wide significant loci associated with plasma galectin-3 levels. One locus harbours the *LGALS3* gene (rs2274273; P = 2.35×10^−188^) and the other locus the *ABO* gene (rs644234; P = 3.65×10^−47^). The variance explained by the *LGALS3* locus was 25.6% and by the *ABO* locus 3.8% and jointly they explained 29.2%. Rs2274273 lies in high linkage disequilibrium with two non-synonymous SNPs (rs4644; r^2^ = 1.0, and rs4652; r^2^ = 0.91) and wet lab follow-up genotyping revealed that both are strongly associated with galectin-3 levels (rs4644; P = 4.97×10^−465^ and rs4652 P = 1.50×10^−421^) and were also associated with *LGALS3* gene-expression. The origins of our associations should be further validated by means of functional experiments.

## Introduction

Galectin-3 (LGALS3) is a lectin and member of the galectin family of carbohydrate binding proteins that have an affinity for beta-galactosides. Galectin-3 plays a role in fibrosis, inflammation, and proliferation [1], [2], [3]. Galectin-3 is secreted into the systemic circulation by unknown mechanisms and is increasingly recognised as a potential biomarker with clinical value. Increased galectin-3 levels have been associated with various diseases, including cancer [4], [5], immunological disorders [6], [7], and cardiovascular traits [8], [9]. Plasma galectin-3 levels are even being considered as a marker of response to cancer treatment [10].

To enhance our knowledge on galectin-3 biology we performed the first genome-wide association study (GWAS) on circulating galectin-3 levels and observed two loci associated with circulating galectin-3 levels. One locus harbours *LGALS3* the gene encoding galectin-3 and the other locus harbours the *ABO* gene which has previously been associated with inflammatory markers, lipids and haematological parameters.

## Results

**Figure 1:**
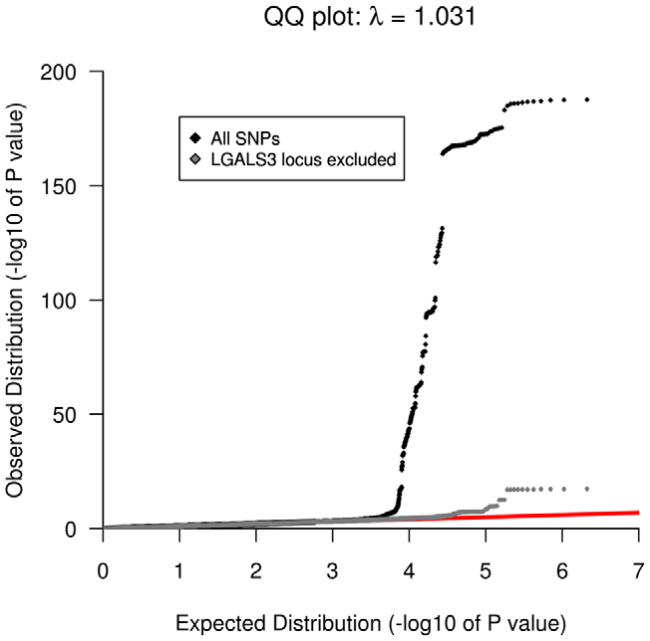
Quantile-quantile plots of observed versus expected p-values for Galectin-3 with and without the *GALS3* locus.

**Figure 2:**
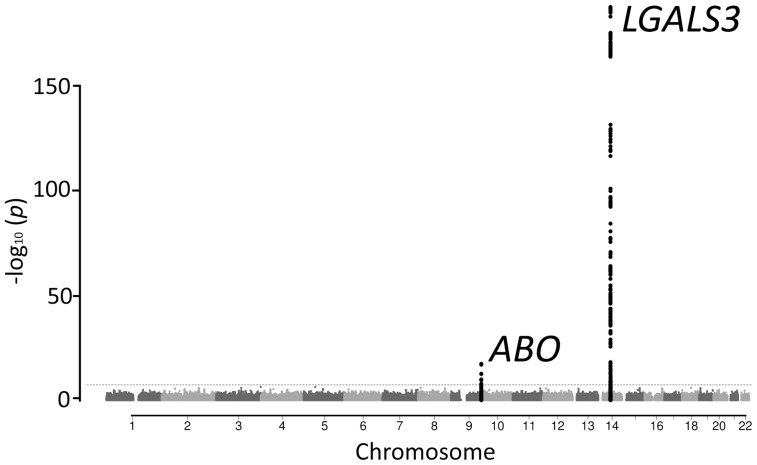
Manhattan plot showing the association of SNPs with circulating galectin-3 levels in a GWAS of 3,776 individuals. The red dotted line marks the threshold for genome-wide significance (P = 5×10^−8^). Two loci reached genome-wide significance.

**Figure 3:**
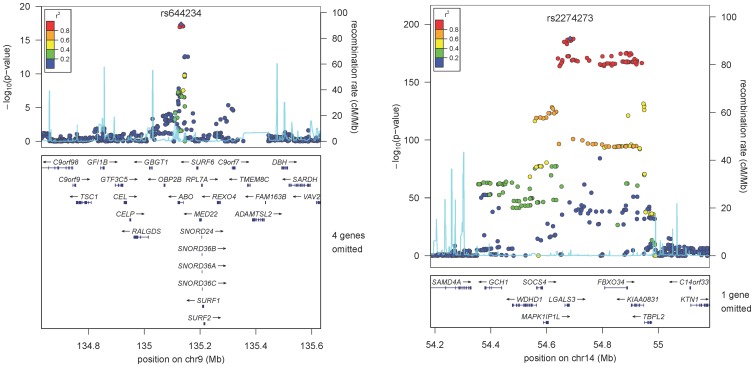
Regional plots at the two significantly associated loci. Horizontal axis indicates chromosomal location and P-values are indicated in the left y-axis. Each plot shows approximately ± 500 kb around each lead SNP and has known gene transcripts annotated at the bottom. The SNPs are colored according to their degree of linkage disequilibrium (*r* ^2^) with the lead SNP which is highlighted with a purple diamond and displayed by rs number and significance level achieved in the discovery analysis.

We performed a GWAS analysis of 2,269,099 genotyped or imputed autosomal SNPs (HapMap 2 build 36 CEU panel) in 3,776 subjects of the PREVEND cohort (**Table 1**, **Table S1**). All included subjects were of European descent. The quantile-quantile plot for association is shown in **Figure 1**. There were 2 loci significantly associated with galectin-3 levels (P<5×10^−8^) and 11 SNPs showing suggestive evidence (P<5×10^−6^ and P>5×10^−8^, **Figure 2**, **Table 2**). We performed further testing of the lead-SNP of the loci using an additional subset of 3,516 independent subjects derived from the PREVEND cohort (**Table 1**). Using inverse-variance fixed effect meta-analysis we combined the evidence. None of the suggestive, but both 2 P<5×10^−8^ loci of the discovery phase, were confirmed in the independent samples (**Table 2**). One locus harbours the *LGALS3* gene and the other locus harbours the *ABO* gene (**Figure 3**). The *LGALS3* locus accounted for 25.6% of the phenotypic variance. The *ABO* locus explained 3.8% and together *LGALS3* and *ABO* explained 29.2% of the phenotypic variance. Of note, common genetic variation explained twice the amount of the variation of circulating galectin-3 levels compared to age, age squared (age^2^), gender, and body mass index combined (11.6%).

**Table 1:** Galectin-3 levels in the PREVEND cohort, indicated for the total population and for the discovery and replication groups.

	Total	Discovery	Follow-up
	N = 7,292	N = 3,776	N = 3,516
Galectin-3, median [IQR]	10.9 [9]–[13]	10.8 [9]–[13]	10.9 [9]–[13]
Galectin-3 (ng/ml), min-max	3.4–233	3.5–233	3.4–200

IQR; inter quartile range.

**Table 2:** Discovery and follow-up genotyping results.

				Discovery		Follow-up		Combined			Annotation	
Chr	SNP	A1/A2	AF	Effect (se)	P-value	Effect (se)	P-value	Effect (se)	P-value	N	Location	Nearest Gene
2	rs17775170	A/G	0.27	−4.79E-02 (0.011)	5.36E-06	3.70E-03 (0.008)	6.218E-01	−1.38E-02 (0.006)	2.43E-02	7284	intronic	SLC9A2
2	rs2165179	A/G	0.33	−4.85E-02 (0.010)	1.47E-06	7.46E-03 (0.015)	6.280E-01	−3.17E-02 (0.008)	1.77E-04	7264	intronic	SCN3A
2	rs7586	T/C	0.03	1.12E-01 (0.025)	7.79E-06	3.75E-03 (0.017)	8.215E-01	3.69E-02 (0.014)	7.61E-03	7278	3′-UTR	COL5A2
3	rs11919628	T/G	0.07	6.11E-02 (0.013)	1.37E-06	9.69E-03 (0.012)	4.218E-01	3.43E-02 (0.009)	8.38E-05	7289	intergenic	FLJ25363, PVRL3-AS1
4	rs930956	C/G	0.33	3.92E-02 (0.009)	7.84E-06	1.34E-02 (0.007)	5.294E-02	2.33E-02 (0.005)	1.89E-05	7277	intronic	ATP8A1
5	rs16902429	G/T	0.29	3.26E-02 (0.007)	4.66E-06	1.04E-02 (0.007)	1.227E-01	2.09E-02 (0.005)	1.86E-05	7287	intergenic	COX7C, MIR4280
6	rs17658562	T/C	0.14	5.29E-02 (0.012)	6.57E-06	−8.91E-03 (0.010)	3.651E-01	1.67E-02 (0.008)	2.67E-02	7268	intergenic	MIR548A1, ID4
8	rs2409784	C/A	0.75	−5.29E-02 (0.011)	1.45E-06	1.10E-03 (0.006)	8.639E-01	−1.26E-02 (0.006)	2.29E-02	7225	intronic	BLK
8	rs4876386	C/T	0.06	6.43E-02 (0.014)	2.51E-06	−4.79E-04 (0.007)	9.482E-01	1.42E-02 (0.007)	2.81E-02	7277	intergenic	MED30, EXT1
**9**	**rs644234**	**G/T**	**0.35**	**−6.05E-02 (0.007)**	**4.52E-18**	**−7.55E-02** **(0.007)**	**2.390E-** **30**	**−6.84E-02 (0.005)**	**3.65E-47**	**7225**	i**ntronic**	**ABO**
11	rs4351827	T/C	0.25	4.98E-02 (0.011)	2.37E-06	4.55E-03 (0.008)	5.655E-01	2.10E-02 (0.006)	9.14E-04	7253	intergenic	CLMP, MIR4493
13	rs9512645	T/C	0.54	−5.31E-02 (0.011)	3.43E-06	6.06E-03 (0.007)	4.185E-01	−1.18E-02 (0.006)	5.97E-02	7281	intergenic	RASL11A, GTF3A
**14**	**rs2274273**	**A/G**	**0.41**	**−1.85E-01 (0.006)**	**2.35E-188**	**NA**	**NA**	**−1.85E-01 (0.006)**	**2.35E-188**	**3776**	**downstream**	**DLGAP5**
**14**	**rs4644**	**A/C**	**0.41**	**−1.85E-01 (0.006)**	**2.06E-187**	**−1.89E-01** **(0.005)**	**3.000E-223**	**−1.87E-01 (0.004)**	**4.97e-465**	**7281**	**exonic**	**LGALS3**
**14**	**rs4652**	**C/A**	**0.43**	**−1.77E-01 (0.006)**	**3.48E-173**	**−1.79E-01** **(0.006)**	**1.160E-202**	**−1.78E-01 (0.004)**	**1.50e-421**	**7292**	**exonic**	**LGALS3**
15	rs17223788	C/T	0.49	5.54E-02 (0.012)	1.65E-06	7.99E-03 (0.006)	2.144E-01	1.93E-02 (0.006)	5.89E-04	7272	intergenic	LOC283688, ARNT2
16	rs8101416	T/A	0.17	4.60E-02 (0.010)	1.94E-06	−8.56E-05 (0.009)	9.928E-01	−2.24E-02 (0.007)	9.52E-04	7257	intergenic	DUS3L, NRTN
19	rs12443973	A/C	0.20	−4.80E-02 (0.010)	2.11E-06	5.84E-03 (0.008)	4.405E-01	−1.35E-02 (0.006)	2.55E-02	7281	intergenic	MAF, MIR548H4

Significant loci (P<5×10^−8^, bold) and suggestive loci (P<5×10^−6^) in the discovery phase that were taken forward for follow-up genotyping. In addition, 2 non-synonymous variants (rs4644 and rs4652) were genotyped. AF: Allele Frequency; (se): standard error.

### Putative causal genetic variants

**Figure 4:**
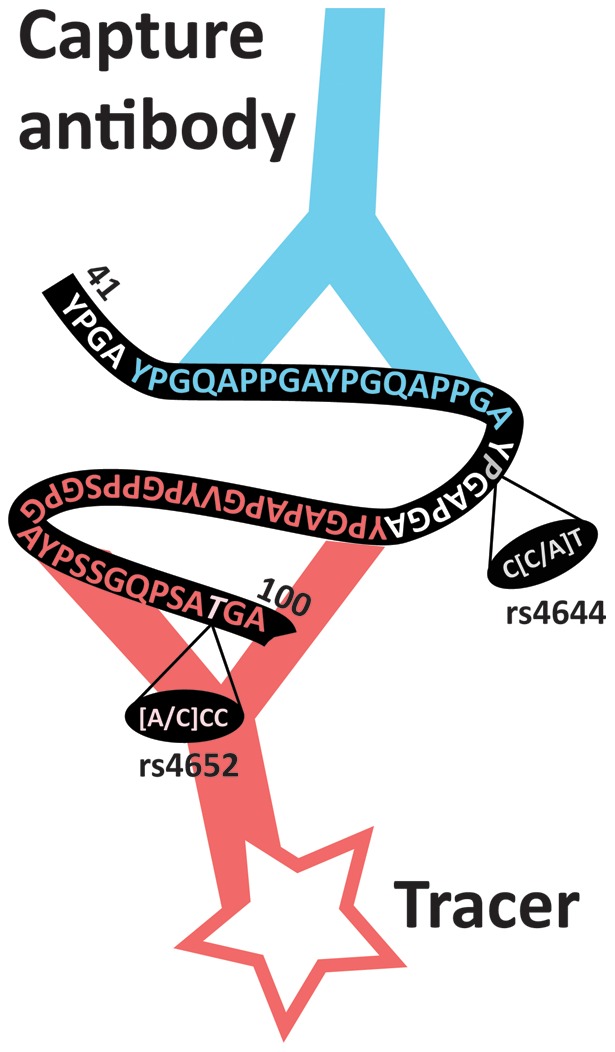
Amino acid sequence (41 to 100) of Galectin-3. The two antibodies used in the Galectin-3 assay recognize epitopes within the N-terminus of the protein (white-colored amino acids). The capture antibody of the galectin-3 assay binds to amino acids number 45 to 62 and the tracer antibody binds to amino acids number 70 to 100. Indicated are the non-synonymous SNPs rs4644 and rs4652.

The lead SNP (rs2274273) of the *LGALS3* locus lies in high LD with two non-synonymous variants (rs4644; r^2^ = 1.0 and rs4652; r^2^ = 0.91). As these variants were not present on our platform and not well imputed we wet-lab genotyped these variants and confirmed their association (**Table 2**). We next considered potential confounding by the specific galectin-3 assay used and noticed the epitopes of the antibodies used are directed against the region harbouring the non-synonymous variant (**Figure 4**). Therefore, this variant might affect the affinity of the antibody and not represent a true difference in circulating galectin-3 levels. We did not find variants in high LD (r^2^>0.8) associated with the lead SNP (rs644234) of the *ABO* locus. Next, we searched for eQTLs in 1,469 samples from peripheral blood for which gene expression levels were obtained using illumine HT12V3 and illumine H8v2 platforms [11]. rs2274273, rs4644, and rs4652 were all associated with *LGALS3* gene expression levels (**Table 3**) and rs2274273 and rs4644 were also the strongest SNP associated with that particular *LGALS3* probe. Finally, to gain further insights we queried the catalogue of published genome wide association studies [12] for our loci and observed no previous associations for the *LGALS3* locus but many previous genome wide associations findings have been reported for the *ABO* locus. Previous SNP associations in or near *ABO* are in high linkage disequilibrium with our lead SNP and include associations with inflammatory markers, lipids and haematological parameters as well as diseases such as cancer and coronary heart disease (**Table S2**).

**Table 3:** Relationship between identified SNPs with expression of *cis*-genes in peripheral blood in 1,469 samples.

Chr	SNP	Position	Gene	Probe	P-Value	FDR
14	rs4644	54673724	LGALS3	5420377	2.42×10^−7^	0
14	rs4652	54674789	LGALS3	5420377	3.10×10^−6^	0
14	rs2274273	54673724	LGALS3	3450685	4.45×10^−6^	0

*cis*-genes: genes within ±1 MB around the lead SNP. FDR: False discovery rate.

**Table 4:** Effect of *LGALS3* genotype on association of plasma galectin-3 levels on mortality.

	All cause				Cardiovascular				Cancer			
Model	HR without rs2274273 (95% CI)	*P*-value	HR with rs2274273 (95% CI)	*P*-value	HR without rs2274273 (95% CI)	*P*-value	HR with rs2274273 (95% CI)	*P*-value	HR without rs2274273 (95% CI)	*P*-value	HR with rs2274273 (95% CI)	*P*-value
Unadjusted	1.46 (1.37–1.56)	0	1.52 (1.43–1.62)	0	1.56 (1.39–1.75)	0	1.60 (1.44–1.78)	0	1.41 (1.28–1.56)	0	1.47 (1.34–1.62)	0
Age and gender adjusted	1.12 (1.03–1.21)	0.007	1.20 (1.09–1.31)	0	1.16 (1.01–1.35)	0.044	1.27 (1.07–1.49)	0.005	1.10 (0.97–1.23)	0.128	1.16 (1.02–1.33)	0.027
Multivariable classical risk factor adjusted*	1.09 (1.01–1.19)	0.036	1.17 (1.06–1.28)	0.001	1.10 (0.94–1.28)	0.228	1.22 (1.03–1.45)	0.02	1.08 (0.96–1.22)	0.222	1.13 (0.99–1.30)	0.067

HR indicates hazard ratio per SD change in plasma galectin-3 level. * the multivariable model included age, sex, hypertension, hypercholesterolemia, diabetes, and smoking.

### Relevance of LGALS3 variant for prognostic value of plasma galectin-3 levels

To study the relevance of the rs2274273 and rs4644 (r^2^ = 1) variant in the *LGALS3* locus for the prognostic value of the galectin-3 assay on mortality in the general population we repeated our earlier reported analyses [13] with and without rs2274273 as a covariate in the model. Knowledge of the genotype did not appear to change the prognostic value of plasma galectin-3 levels (**Table 4**).

## Discussion

We report the first genetic association study on galectin-3 levels and identified 2 genome-wide significant loci; one including the galectin-3 encoding gene (*LGALS3*) and the other gene being *ABO*.

Galectin-3 is a member of the galectin family that comprises of lectins with affinity for beta-galactosidases containing carbohydrates. The galectin gene family is evolutionarily ancient and can be found in vertebrates, invertebrates, and even in protists suggesting an important role in biology [14]. All galectins have a carbohydrate-recognition domain (CRD) consisting of many conserved sequence elements and each galectin has an individual carbohydrate-binding preference [15]. Galectin-3 is an unique galectin as it contains a non-lectin N-terminal region which is connected to the CRD. Galectin-3 is therefore referred to as a chimera-like galectin [1]. Galectin-3 does not contain a signal sequence and is primarily localised within the cytoplasm. It can be externalized by a mechanism independent of the endoplasmic reticulum (ER)-Golgi complex [16], [17]. Galectin-3 has high affinity for lactose and N-acetyllactosamine but can also interact with a wide array of other carbohydrates, membrane and extracellular matrix proteins [18]. Upon ligand binding, galectin-3 (and its ligands) forms cross-links, is involved in strengthening cell-cell interactions, and is associated with stiffening of the extracellular matrix and fibrogenesis. Galectin-3 has been shown to play a role in inflammatory diseases, cancer and heart failure [3], [15], [19], [20]. Little is known about the regulation of galectin-3. The galectin-3 promoter contains several responsive elements, including Sp-1, AP-1 and cAMP responsive elements [21].

We now report the first 2 genome wide associations with circulating galectin-3 levels. The strongest locus is within the *LGALS3* gene. The lead SNP (rs2274273) is in full LD with two non-synonymous SNPs (rs4644 and rs4652) which were confirmed by follow-up genotyping. Both rs2274273 and rs4644 affected *LGALS3* gene-expression providing a potential explanation for the observed effect. In the current study we also tested whether knowledge of the lead variant in the *LGALS3* gene might obscure the association of plasma galectin-3 levels with outcome but it did not alter our previously published associations further supporting a true effect of these variant on galectin-3 [13]. However, some note of caution is warranted. Associations of coding SNPs (e.g. rs4644 and rs4652) that structurally change the properties of its encoded protein can give rise to false positive associations when that protein is also the phenotype under investigation. The non-synonymous SNPs identified in our study also lies within or near the epitopes of the antibodies used for the galectin-3 assay (**Figure 4**). These antibodies might have different affinities for the amino acid change and therefore this association could also be artifactual. Interference of antibody based assays with epitopes directed against regions harbouring non-synonymous variants are not novel and have previously been reported for the *NPPA-NPPB* locus when ANP levels were measured [22]. Although gene-expression analyses and association with outcome are suggesting a true effect, additional work will be required to define the precise mechanisms of our reported association at the *LGALS3* locus.

Our second genome wide locus is the *ABO* locus. The *ABO* locus is becoming an increasingly complex and pleiotropic locus. Variants in *ABO*, and in high LD with our lead SNP (rs644234), have been associated by genome wide association studies with various blood measured traits and diseases. This includes several inflammatory markers, lipids, hematological parameters, cancer, inflammatory diseases, and cardiovascular diseases (**Table S2**). Interestingly, galectin-3 levels also are associated with many of these conditions. Galectin-3 can indeed bind to polysaccharides of the ABO epitopes and even more strongly to the A- or B-histo-blood group epitopes versus the O group [23]. However, this does not explain how the *ABO* gene variant affects circulating galectin-3 levels.

In summary, we performed a GWAS on plasma galectin-3 levels and identified two genome wide significant loci, one including the *LGALS3* gene and the other the *ABO* gene. The origins of these associations should be further validated by means of functional experiments.

## Materials and Methods

### Study population

We studied subjects included in the PREVEND cohort. The PREVEND cohort has been described in detail elsewhere [13], [24], [25]. In brief, 8,592 subjects were enrolled in the PREVEND cohort in 1997–1998. Subjects were asked to refrain from eating and drinking prior to their visit (fasting) in the outpatient clinic (between 8:00 a. um and 1:00 pm) and blood samples were drawn and stored at −80C. The PREVEND study was approved by the local medical Ethical Committee, and is conducted in accordance with the guidelines of the Declaration of Helsinki. All subjects provided written informed consent.

### Galectin-3 Measurements

For 7,968 subjects plasma was available to measure plasma galectin-3 levels [13]. The galectin-3 assay is an enzyme-linked immunosorbent assay (BG Medicine, Inc., Waltham, USA). This assay quantitatively measures the concentration of human galectin-3 levels in EDTA plasma. This assay has high sensitivity (lower limit of detection 1.13 ng/mL) and exhibits no cross reactivity with collagens or other members of the galectin family [26]. Commonly used medication like ACE-inhibitors, beta blockers, spironolactone, furosemide, acetylsalicylic acid, warfarin, coumarines, and digoxin have no interference with the assay [26]. All samples were assayed in duplicate. Two standard controls were included in all runs: a lower control (expected value: 13.0–23.1 ng/mL) and a higher control (expected value: 48.9–81.5 ng/mL). The average lower control results were 16.65±1.13 (coefficient of variance: 6.8%), and the average higher control results were 68.17±3.20 (coefficient of variance: 4.7%).

### Genotyping, Quality control & Imputation

Genotyping in 4,016 of the total number of participants in PREVEND was carried out using Illumina HumanCytoSNP-12 arrays. SNPs were called using Illumina Genome Studio software. Forty-seven subjects were excluded from analyses because call rates were <0.95. Another 65 subjects were excluded because they were closely related as judged based on Identity-By-Descent estimation using PLINK v1.07. Population structure was assessed using PCA based on 16,842 independent SNPs. Based on this analysis, an additional 2 samples were excluded that diverged from the mean with at least 3 standard deviations (Z-score >3) for the first 5 PCAs. Another 35 subjects were excluded based on sex inconsistencies. We excluded samples with a genetic similarity >0.1. Of 87 subjects no phenotype was available because of missing plasma samples for assessment of Galectin-3. As a consequence 3,776 (1,927 males, 1,849 females) were available for GWAS analysis. SNPs were excluded with a minor allele frequency of <0.01, call rate <0.95, or deviation from Hardy Weinberg equilibrium (P<1×10^−5^). Genome wide genotype imputation was performed using Beagle v. 3.3.1 [27], 232,571 genotyped SNPs were imputed up to 2,269,099 autosomal SNPs with NCBI build 36 of Phase II HapMap CEU data (release 22) as reference panel. Replication genotyping was performed by KBiosciences (KBiosciences, Herts, UK) utilizing the SNPline system in an additional 3,516 independent subjects of the PREVEND study.

### Gene-expression analyses

We investigated whether each of the associated variants had an effect on gene expression levels by mapping *cis*-expression quantitative trait loci (*cis*-eQTL) in 1,469 samples from peripheral blood, for which gene expression level measurements were obtained using Illumina HT12v3 and Illumina H8v2 platforms [11]. Since the genotypes were imputed using the CEU population of HapMap 2 release 24 as reference, eQTL effects were tested using the imputation dosage values. Effects for SNPs (MAF >5%, HWE >0.001) were considered *cis*-eQTLs when the distance between the SNP and the midpoint position of the probe was smaller than 1 MB. As multiple testing correction, we controlled the false discovery rate (FDR) at 0.05, by comparing observed p-values to the null distribution obtained from permuting the expression phenotype labels relative to genotype labels 100 times. We also determined the top eQTL SNP for each given probe and tested whether the GWAS SNP had an independent effect on the associated gene expression probe after removing the effect of the top eQTL SNP.

### Statistical analysis

Galectin-3 was non-normally distributed and was log transformed before regression analyses. We calculated residuals of galectin-3 levels after adjustment for age, age^2^, and gender. GWAS analyses were performed on residuals using an additive genetic model in PLINK (v 1.07) [28]. The most significant (P<5×10^−8^) SNPs (lead SNP) at each locus was taken forward for further testing. The explained variance of the significant associations was analysed using the directly genotyped variants from the replication stage. Fixed-effect meta-analysis was performed using the variance weighting method of the METAL software package to calculate the overall p-value. The Cox proportional-hazards model was used to calculate the hazard ratio and 95% confidence intervals (CI) of galectin-3. Based on our previous work, sequential models were fitted without and with the SNP of interest [13]. The first model including no covariates (unadjusted) and the second model adjusted for age and gender and the third model adjusted for: age, gender, previous myocardial infarction, previous stroke, hypertension, hypercholesterolemia and diabetes. The assumptions underlying the proportional hazards model were tested and found valid. Analyses were performed using STATA version 11.0 for Windows software (StataCorp LP, College Station, TX, USA).

## Supporting Information

Table S1 **SNPs significantly associated with galectin-3 levels at the Discovery stage.**
(DOC)

Table S2 **Previous reported traits with genome wide associations at the ABO locus.**
(DOC)
