# Supplementary material for: A Genome-Wide Association Study of Circulating Galectin-3
Source: PLoS One. 2012 Oct 9;7(10):e47385. doi: 10.1371/journal.pone.0047385 (PMC3467202; doi:10.1371/journal.pone.0047385)
Supplement: Table S1 — SNPs significantly associated with galectin-3 levels at the Discovery stage. (DOC) [file pone.0047385.s001.doc]

**Table S1**. SNPs significantly associated with galectin-3 levels at the Discovery stage.

| CHR | SNP | BP | A1/A2 | AF | Effect (se) | P-value |
| --- | --- | --- | --- | --- | --- | --- |
| 9 | rs8176740 | 135121296 | T/A | 0.26 | 4.12E-02(0.008) | 5.10E-08 |
| 9 | rs8176732 | 135122128 | G/A | 0.26 | 4.09E-02(0.008) | 5.51E-08 |
| 9 | rs8176728 | 135122384 | C/G | 0.26 | 4.10E-02(0.008) | 5.51E-08 |
| 9 | rs2073825 | 135122528 | T/A | 0.26 | 4.10E-02(0.008) | 5.75E-08 |
| 9 | rs8176717 | 135122848 | T/G | 0.26 | 4.11E-02(0.008) | 5.50E-08 |
| 9 | rs8176714 | 135122992 | A/G | 0.26 | 4.11E-02(0.008) | 5.66E-08 |
| 9 | rs512770 | 135123328 | A/G | 0.17 | 6.79E-02(0.012) | 1.41E-08 |
| 9 | rs641959 | 135123520 | C/A | 0.27 | 4.19E-02(0.008) | 4.36E-08 |
| 9 | rs641943 | 135123536 | G/A | 0.27 | 4.18E-02(0.008) | 4.56E-08 |
| 9 | rs514708 | 135123568 | T/C | 0.27 | 4.19E-02(0.008) | 4.57E-08 |
| 9 | rs517414 | 135123856 | A/G | 0.26 | 4.27E-02(0.008) | 4.39E-08 |
| 9 | rs626035 | 135124816 | G/T | 0.26 | 4.27E-02(0.008) | 4.41E-08 |
| 9 | rs547643 | 135124864 | T/C | 0.26 | 4.27E-02(0.008) | 4.48E-08 |
| 9 | rs549331 | 135125024 | G/C | 0.26 | 4.27E-02(0.008) | 4.50E-08 |
| 9 | rs549446 | 135125056 | T/C | 0.26 | 4.27E-02(0.008) | 4.47E-08 |
| 9 | rs624601 | 135125184 | A/G | 0.26 | 4.27E-02(0.008) | 4.44E-08 |
| 9 | rs613423 | 135125296 | A/G | 0.26 | 4.27E-02(0.008) | 4.43E-08 |
| 9 | rs574347 | 135125472 | C/T | 0.26 | 4.28E-02(0.008) | 4.55E-08 |
| 9 | rs579483 | 135126016 | A/T | 0.26 | 4.28E-02(0.008) | 4.71E-08 |
| 9 | rs579622 | 135126064 | A/G | 0.26 | 4.27E-02(0.008) | 4.86E-08 |
| 9 | rs493211 | 135126336 | A/G | 0.27 | 4.31E-02(0.008) | 5.78E-08 |
| 9 | rs688976 | 135126592 | A/C | 0.26 | 4.27E-02(0.008) | 4.89E-08 |
| 9 | rs687621 | 135126880 | G/A | 0.34 | -6.02E-02(0.007) | 1.09E-17 |
| 9 | rs687289 | 135126928 | A/G | 0.34 | -6.01E-02(0.007) | 1.10E-17 |
| 9 | rs8176693 | 135127472 | T/C | 0.06 | -7.63E-02(0.014) | 7.57E-08 |
| 9 | rs657152 | 135129088 | A/C | 0.35 | -5.88E-02(0.007) | 6.01E-18 |
| 9 | rs644234^†^ | 135132032 | G/T | 0.35 | -6.05E-02(0.007) | 4.52E-18 |
| 9 | rs514659 | 135132032 | C/A | 0.34 | -6.13E-02(0.007) | 5.99E-18 |
| 9 | rs643434 | 135132176 | A/G | 0.35 | -6.08E-02(0.007) | 5.65E-18 |
| 9 | rs545971 | 135133200 | T/C | 0.33 | -6.13E-02(0.007) | 8.82E-18 |
| 9 | rs612169 | 135133264 | G/A | 0.33 | -6.14E-02(0.007) | 8.92E-18 |
| 9 | rs674302 | 135136480 | A/T | 0.33 | -6.15E-02(0.007) | 8.37E-18 |
| 9 | rs505922 | 135139056 | C/T | 0.33 | -6.15E-02(0.007) | 8.43E-18 |
| 9 | rs529565 | 135139328 | C/T | 0.33 | -6.15E-02(0.007) | 8.57E-18 |
| 9 | rs630014 | 135139536 | A/G | 0.52 | 6.36E-02(0.011) | 2.81E-08 |
| 9 | rs579459 | 135143984 | C/T | 0.21 | -5.04E-02(0.008) | 1.91E-10 |
| 9 | rs9411488 | 135144064 | T/G | 0.24 | -1.34E-01(0.018) | 3.20E-13 |
| 9 | rs649129 | 135144128 | T/C | 0.22 | -5.09E-02(0.008) | 1.74E-10 |
| 9 | rs495828 | 135144688 | T/G | 0.22 | -5.10E-02(0.008) | 1.94E-10 |
| 9 | rs635634 | 135144816 | T/C | 0.19 | -5.79E-02(0.009) | 2.68E-10 |
| 9 | rs13298002 | 135144944 | A/G | 0.08 | -2.23E-01(0.035) | 1.22E-10 |
| 9 | rs9411491 | 135145840 | C/T | 0.24 | -1.42E-01(0.019) | 2.47E-13 |
| 9 | rs7025162 | 135156160 | T/C | 0.24 | -1.42E-01(0.019) | 2.81E-13 |
| 14 | rs7156891 | 54271256 | G/A | 0.14 | -6.11E-02(0.01) | 2.47E-09 |
| 14 | rs7156787 | 54271432 | A/G | 0.14 | -6.12E-02(0.01) | 2.38E-09 |
| 14 | rs7144602 | 54355336 | G/T | 0.35 | -1.27E-01(0.008) | 1.27E-62 |
| 14 | rs9323272 | 54358672 | A/G | 0.35 | -1.27E-01(0.008) | 1.24E-62 |
| 14 | rs7141685 | 54360220 | T/G | 0.35 | -1.27E-01(0.008) | 1.24E-62 |
| 14 | rs10135789 | 54361768 | G/A | 0.35 | -1.27E-01(0.008) | 1.16E-62 |
| 14 | rs7151058 | 54365688 | G/C | 0.23 | -1.22E-01(0.008) | 5.47E-51 |
| 14 | rs2057368 | 54373760 | A/G | 0.21 | -1.22E-01(0.008) | 2.42E-53 |
| 14 | rs2057369 | 54373812 | A/G | 0.21 | -1.22E-01(0.008) | 2.45E-53 |
| 14 | rs2145945 | 54373832 | A/G | 0.33 | -1.25E-01(0.007) | 4.73E-64 |
| 14 | rs17128004 | 54375992 | A/C | 0.21 | -1.22E-01(0.008) | 2.16E-53 |
| 14 | rs10483639 | 54376208 | C/G | 0.21 | -1.22E-01(0.008) | 2.15E-53 |
| 14 | rs7142517 | 54376552 | A/C | 0.51 | 1.22E-01(0.014) | 1.71E-18 |
| 14 | rs841 | 54380240 | A/G | 0.21 | -1.22E-01(0.008) | 2.20E-53 |
| 14 | rs752688 | 54381320 | T/C | 0.21 | -1.22E-01(0.008) | 2.21E-53 |
| 14 | rs10131232 | 54385656 | A/G | 0.32 | -1.23E-01(0.007) | 8.27E-64 |
| 14 | rs10133662 | 54386020 | G/A | 0.32 | -1.23E-01(0.007) | 8.12E-64 |
| 14 | rs4411417 | 54390312 | C/T | 0.21 | -1.22E-01(0.008) | 2.16E-53 |
| 14 | rs2878168 | 54390536 | A/G | 0.21 | -1.21E-01(0.008) | 2.57E-53 |
| 14 | rs7155309 | 54392600 | C/T | 0.21 | -1.22E-01(0.008) | 2.12E-53 |
| 14 | rs8007201 | 54394600 | G/A | 0.32 | -1.23E-01(0.007) | 7.65E-64 |
| 14 | rs12587434 | 54395332 | G/T | 0.21 | -1.22E-01(0.008) | 1.86E-53 |
| 14 | rs12589758 | 54395612 | T/A | 0.21 | -1.22E-01(0.008) | 2.02E-53 |
| 14 | rs9671371 | 54398384 | T/C | 0.31 | -1.20E-01(0.007) | 2.81E-63 |
| 14 | rs9671455 | 54398832 | G/C | 0.21 | -1.23E-01(0.008) | 7.18E-54 |
| 14 | rs2183081 | 54406500 | G/A | 0.35 | -9.37E-02(0.007) | 1.03E-38 |
| 14 | rs10498472 | 54424620 | G/T | 0.08 | -6.71E-02(0.012) | 5.55E-08 |
| 14 | rs998259 | 54424780 | T/C | 0.39 | 1.01E-01(0.014) | 2.58E-13 |
| 14 | rs17128052 | 54426276 | C/G | 0.21 | -1.35E-01(0.008) | 1.49E-62 |
| 14 | rs7147286 | 54428416 | A/G | 0.35 | -9.57E-02(0.007) | 5.11E-42 |
| 14 | rs7147201 | 54428628 | G/A | 0.21 | -1.35E-01(0.008) | 8.41E-63 |
| 14 | rs17832263 | 54429572 | A/G | 0.08 | -6.97E-02(0.013) | 2.35E-08 |
| 14 | rs3783641 | 54429888 | A/T | 0.21 | -1.35E-01(0.008) | 4.52E-63 |
| 14 | rs3783642 | 54429952 | C/T | 0.43 | -1.00E-01(0.006) | 2.95E-53 |
| 14 | rs8017210 | 54431584 | A/G | 0.20 | -1.38E-01(0.008) | 2.54E-63 |
| 14 | rs3759664 | 54441328 | T/C | 0.20 | -1.37E-01(0.008) | 8.49E-63 |
| 14 | rs2878172 | 54443420 | G/A | 0.43 | -1.03E-01(0.007) | 1.76E-55 |
| 14 | rs11847771 | 54444352 | A/T | 0.20 | -1.36E-01(0.008) | 2.84E-62 |
| 14 | rs11621165 | 54445176 | A/G | 0.21 | -1.37E-01(0.008) | 1.69E-62 |
| 14 | rs8007267 | 54448740 | T/C | 0.20 | -1.37E-01(0.008) | 4.22E-63 |
| 14 | rs11158028 | 54450820 | T/C | 0.20 | -1.37E-01(0.008) | 2.50E-63 |
| 14 | rs8022453 | 54451540 | A/G | 0.21 | -1.36E-01(0.008) | 9.02E-63 |
| 14 | rs943912 | 54455076 | G/C | 0.21 | -1.38E-01(0.008) | 1.05E-64 |
| 14 | rs10140164 | 54475872 | G/A | 0.44 | -1.13E-01(0.007) | 5.71E-64 |
| 14 | rs8020545 | 54478820 | A/T | 0.33 | -9.66E-02(0.007) | 2.95E-42 |
| 14 | rs8019390 | 54478832 | G/C | 0.33 | -9.67E-02(0.007) | 2.60E-42 |
| 14 | rs7146285 | 54482620 | C/T | 0.35 | -1.01E-01(0.007) | 3.73E-48 |
| 14 | rs8009997 | 54491660 | G/T | 0.33 | -9.64E-02(0.007) | 3.77E-42 |
| 14 | rs8012152 | 54495316 | C/T | 0.35 | -1.00E-01(0.007) | 4.35E-48 |
| 14 | rs11627963 | 54496296 | G/C | 0.20 | -1.33E-01(0.008) | 1.50E-60 |
| 14 | rs9285583 | 54501184 | C/T | 0.35 | -1.00E-01(0.007) | 6.09E-48 |
| 14 | rs17832269 | 54501840 | T/C | 0.34 | -9.74E-02(0.007) | 3.38E-42 |
| 14 | rs943914 | 54509816 | A/C | 0.34 | -9.66E-02(0.007) | 4.50E-44 |
| 14 | rs7151670 | 54522508 | G/C | 0.34 | -9.66E-02(0.007) | 4.58E-44 |
| 14 | rs8018800 | 54523056 | G/A | 0.36 | -9.97E-02(0.007) | 1.27E-49 |
| 14 | rs8019220 | 54523220 | T/C | 0.36 | -9.99E-02(0.007) | 1.07E-49 |
| 14 | rs8022049 | 54532620 | C/T | 0.34 | -9.66E-02(0.007) | 5.06E-44 |
| 14 | rs17253633 | 54539668 | C/T | 0.20 | -1.30E-01(0.008) | 1.21E-58 |
| 14 | rs2878174 | 54540376 | C/T | 0.45 | -1.16E-01(0.007) | 4.53E-69 |
| 14 | rs4335711 | 54540612 | C/G | 0.34 | -9.72E-02(0.007) | 2.26E-44 |
| 14 | rs7153937 | 54540624 | G/A | 0.36 | -1.01E-01(0.007) | 3.96E-50 |
| 14 | rs3783646 | 54541636 | C/T | 0.34 | -9.72E-02(0.007) | 2.23E-44 |
| 14 | rs2183085 | 54542140 | C/A | 0.36 | -1.01E-01(0.007) | 1.88E-50 |
| 14 | rs17128120 | 54544348 | T/C | 0.36 | -1.01E-01(0.007) | 1.78E-50 |
| 14 | rs10139354 | 54548352 | T/C | 0.36 | -1.01E-01(0.007) | 1.54E-50 |
| 14 | rs1187883 | 54557984 | T/C | 0.43 | -1.55E-01(0.007) | 3.28E-117 |
| 14 | rs1187882 | 54558496 | C/G | 0.36 | -1.29E-01(0.007) | 3.22E-76 |
| 14 | rs1209087 | 54562968 | C/T | 0.43 | -1.51E-01(0.006) | 7.78E-120 |
| 14 | rs1187878 | 54563100 | T/C | 0.37 | -1.25E-01(0.007) | 1.78E-77 |
| 14 | rs1201378 | 54563160 | T/C | 0.43 | -1.51E-01(0.006) | 7.83E-120 |
| 14 | rs1212641 | 54565880 | G/C | 0.22 | -1.14E-01(0.008) | 8.07E-47 |
| 14 | rs1952438 | 54567696 | A/G | 0.15 | -7.24E-02(0.009) | 1.90E-15 |
| 14 | rs1187874 | 54567852 | A/G | 0.22 | -1.13E-01(0.008) | 6.51E-47 |
| 14 | rs1187873 | 54570004 | C/T | 0.22 | -1.13E-01(0.008) | 6.05E-47 |
| 14 | rs11851178 | 54571880 | G/A | 0.36 | -1.23E-01(0.006) | 3.94E-78 |
| 14 | rs17128132 | 54576512 | T/C | 0.37 | -1.24E-01(0.007) | 3.75E-78 |
| 14 | rs8008134 | 54577272 | C/T | 0.42 | -1.49E-01(0.006) | 2.78E-120 |
| 14 | rs7149965 | 54577944 | A/G | 0.37 | -1.24E-01(0.007) | 3.45E-78 |
| 14 | rs7150145 | 54578012 | A/G | 0.37 | -1.24E-01(0.007) | 3.78E-78 |
| 14 | rs17128136 | 54579864 | A/G | 0.12 | -1.36E-01(0.018) | 4.00E-14 |
| 14 | rs1952085 | 54582024 | A/C | 0.36 | -1.23E-01(0.006) | 6.11E-78 |
| 14 | rs2340943 | 54587760 | T/C | 0.22 | -1.13E-01(0.008) | 2.97E-47 |
| 14 | rs2251366 | 54587908 | G/A | 0.42 | -1.48E-01(0.006) | 1.41E-119 |
| 14 | rs1002054 | 54598664 | C/A | 0.42 | -1.50E-01(0.006) | 3.85E-120 |
| 14 | rs3742564 | 54599872 | C/T | 0.37 | -1.27E-01(0.007) | 2.72E-81 |
| 14 | rs17128143 | 54603340 | G/C | 0.06 | -1.76E-01(0.016) | 1.87E-28 |
| 14 | rs7144652 | 54607472 | A/G | 0.42 | -1.53E-01(0.006) | 5.49E-124 |
| 14 | rs13379159 | 54610552 | T/C | 0.43 | -1.53E-01(0.006) | 6.26E-124 |
| 14 | rs17253660 | 54611120 | T/G | 0.15 | -7.90E-02(0.009) | 8.28E-17 |
| 14 | rs749053 | 54614228 | C/T | 0.15 | -8.02E-02(0.009) | 2.54E-17 |
| 14 | rs873061 | 54616080 | C/T | 0.30 | -1.24E-01(0.007) | 2.61E-70 |
| 14 | rs7157678 | 54618492 | G/C | 0.28 | -1.33E-01(0.007) | 6.00E-78 |
| 14 | rs8004416 | 54619304 | G/A | 0.42 | -1.64E-01(0.007) | 4.25E-129 |
| 14 | rs8012156 | 54622252 | T/G | 0.42 | -1.56E-01(0.006) | 5.48E-128 |
| 14 | rs17128156 | 54622972 | A/G | 0.05 | -1.70E-01(0.016) | 2.64E-26 |
| 14 | rs11625204 | 54624672 | G/A | 0.23 | -1.14E-01(0.008) | 4.36E-49 |
| 14 | rs8019978 | 54627036 | A/G | 0.45 | -1.57E-01(0.006) | 6.26E-124 |
| 14 | rs2147966 | 54628588 | G/C | 0.15 | -8.43E-02(0.01) | 2.08E-18 |
| 14 | rs2147965 | 54629056 | C/G | 0.15 | -8.41E-02(0.01) | 2.60E-18 |
| 14 | rs9671895 | 54629416 | C/T | 0.29 | -1.33E-01(0.007) | 5.30E-78 |
| 14 | rs9806061 | 54630884 | T/C | 0.15 | -8.54E-02(0.01) | 7.21E-19 |
| 14 | rs2340921 | 54633456 | G/A | 0.45 | -1.58E-01(0.006) | 3.60E-125 |
| 14 | rs2147964 | 54634148 | G/T | 0.45 | -1.58E-01(0.006) | 3.47E-125 |
| 14 | rs2147963 | 54634224 | C/T | 0.23 | -1.15E-01(0.008) | 5.36E-49 |
| 14 | rs11622128 | 54634280 | T/G | 0.28 | -1.33E-01(0.007) | 5.17E-78 |
| 14 | rs11626816 | 54634392 | C/T | 0.28 | -1.33E-01(0.007) | 4.37E-78 |
| 14 | rs11627884 | 54634920 | C/T | 0.28 | -1.33E-01(0.007) | 3.72E-78 |
| 14 | rs10147667 | 54640600 | C/T | 0.42 | -1.78E-01(0.006) | 2.89E-171 |
| 14 | rs17253695 | 54644364 | T/A | 0.32 | -1.44E-01(0.007) | 2.32E-97 |
| 14 | rs8007944 | 54646568 | C/A | 0.43 | -1.72E-01(0.006) | 1.26E-164 |
| 14 | rs17128183 | 54649264 | G/A | 0.43 | -1.73E-01(0.006) | 1.03E-165 |
| 14 | rs8009670 | 54653232 | A/G | 0.43 | -1.74E-01(0.006) | 1.99E-167 |
| 14 | rs17253702 | 54653636 | A/G | 0.18 | -1.42E-01(0.009) | 1.27E-60 |
| 14 | rs2147961 | 54655136 | C/T | 0.43 | -1.74E-01(0.006) | 5.04E-168 |
| 14 | rs7158989 | 54655312 | T/C | 0.10 | -1.39E-01(0.011) | 2.84E-36 |
| 14 | rs8007614 | 54656328 | C/T | 0.43 | -1.75E-01(0.006) | 2.66E-169 |
| 14 | rs8007620 | 54656340 | C/T | 0.43 | -1.75E-01(0.006) | 8.38E-170 |
| 14 | rs3825613 | 54662924 | C/A | 0.41 | -1.84E-01(0.006) | 1.30E-185 |
| 14 | rs1009977 | 54672756 | G/T | 0.41 | -1.82E-01(0.006) | 8.74E-184 |
| 14 | rs1009978 | 54672816 | G/C | 0.41 | -1.85E-01(0.006) | 1.95E-186 |
| 14 | rs4644 | 54674688 | A/C | 0.41 | -1.85E-01(0.006) | 2.06E-187 |
| 14 | rs4652 | 54674788 | C/A | 0.43 | -1.77E-01(0.006) | 3.49E-173 |
| 14 | rs8004787 | 54677120 | T/C | 0.43 | -1.77E-01(0.006) | 3.52E-173 |
| 14 | rs2075601 | 54678988 | T/C | 0.43 | -1.78E-01(0.006) | 4.30E-173 |
| 14 | rs2075602 | 54679528 | G/A | 0.43 | -1.77E-01(0.006) | 2.60E-173 |
| 14 | rs2075603 | 54679708 | A/C | 0.41 | -1.85E-01(0.006) | 1.39E-187 |
| 14 | rs8013027 | 54680692 | G/C | 0.41 | -1.85E-01(0.006) | 8.75E-187 |
| 14 | rs10498475 | 54682232 | T/C | 0.08 | -1.70E-01(0.012) | 6.89E-47 |
| 14 | rs2274273 | 54684388 | A/G | 0.41 | -1.85E-01(0.006) | 2.35E-188 |
| 14 | rs2340931 | 54686688 | G/T | 0.41 | -1.86E-01(0.006) | 1.14E-186 |
| 14 | rs11622740 | 54687596 | A/G | 0.41 | -1.85E-01(0.006) | 4.03E-188 |
| 14 | rs15870 | 54689064 | G/A | 0.41 | -1.85E-01(0.006) | 1.03E-187 |
| 14 | rs10140857 | 54689364 | C/G | 0.41 | -1.87E-01(0.006) | 4.09E-187 |
| 14 | rs3818451 | 54690868 | G/C | 0.44 | -1.78E-01(0.006) | 1.29E-169 |
| 14 | rs7159808 | 54694080 | T/G | 0.43 | -1.77E-01(0.006) | 4.30E-170 |
| 14 | rs2296489 | 54694728 | C/T | 0.32 | -1.45E-01(0.007) | 1.15E-101 |
| 14 | rs6573007 | 54695692 | G/T | 0.43 | -1.78E-01(0.006) | 3.27E-170 |
| 14 | rs8011834 | 54695728 | C/A | 0.41 | -1.87E-01(0.006) | 3.10E-188 |
| 14 | rs10134800 | 54704960 | G/A | 0.43 | -1.76E-01(0.006) | 1.77E-169 |
| 14 | rs10134983 | 54705076 | T/C | 0.09 | -1.51E-01(0.011) | 6.31E-40 |
| 14 | rs8018110 | 54711468 | T/C | 0.43 | -1.75E-01(0.006) | 6.73E-169 |
| 14 | rs10144326 | 54713660 | G/A | 0.09 | -1.52E-01(0.011) | 7.57E-41 |
| 14 | rs8003961 | 54721304 | G/A | 0.09 | -1.42E-01(0.011) | 1.58E-37 |
| 14 | rs7159490 | 54724160 | T/C | 0.09 | -1.51E-01(0.011) | 6.27E-40 |
| 14 | rs6573008 | 54724836 | A/C | 0.10 | -1.60E-01(0.011) | 2.20E-44 |
| 14 | rs2274271 | 54725444 | T/C | 0.09 | -1.52E-01(0.011) | 6.50E-41 |
| 14 | rs3759667 | 54728176 | G/A | 0.32 | -1.44E-01(0.007) | 1.47E-100 |
| 14 | rs17671923 | 54733672 | G/A | 0.09 | -1.62E-01(0.012) | 2.57E-43 |
| 14 | rs17739689 | 54735028 | A/T | 0.07 | -4.52E-01(0.027) | 2.45E-62 |
| 14 | rs10147434 | 54744600 | T/C | 0.43 | -1.76E-01(0.006) | 9.90E-172 |
| 14 | rs4040064 | 54747840 | T/G | 0.33 | -1.42E-01(0.007) | 1.09E-97 |
| 14 | rs7160411 | 54748784 | T/C | 0.09 | -1.43E-01(0.011) | 2.15E-37 |
| 14 | rs6573009 | 54752076 | G/T | 0.44 | -1.75E-01(0.006) | 1.98E-169 |
| 14 | rs1952087 | 54752964 | C/A | 0.44 | -1.75E-01(0.006) | 1.39E-169 |
| 14 | rs11845284 | 54753660 | C/T | 0.11 | -1.19E-01(0.01) | 1.21E-29 |
| 14 | rs7141706 | 54756352 | G/C | 0.09 | -1.56E-01(0.012) | 6.45E-40 |
| 14 | rs751059 | 54760280 | G/C | 0.10 | -2.23E-01(0.014) | 3.10E-55 |
| 14 | rs9919921 | 54765256 | G/A | 0.33 | -1.42E-01(0.007) | 3.47E-97 |
| 14 | rs17672364 | 54775656 | T/G | 0.33 | -1.42E-01(0.007) | 2.32E-97 |
| 14 | rs17672376 | 54777116 | T/C | 0.09 | -1.58E-01(0.012) | 5.84E-39 |
| 14 | rs8013528 | 54784592 | T/A | 0.11 | -1.15E-01(0.01) | 5.41E-28 |
| 14 | rs8009718 | 54787132 | G/A | 0.11 | -1.15E-01(0.01) | 3.19E-28 |
| 14 | rs8003311 | 54791028 | T/C | 0.04 | -9.32E-01(0.047) | 5.49E-85 |
| 14 | rs6573010 | 54791344 | C/T | 0.44 | -1.73E-01(0.006) | 3.11E-166 |
| 14 | rs10467798 | 54794680 | T/C | 0.44 | -1.73E-01(0.006) | 1.18E-165 |
| 14 | rs2341621 | 54796176 | T/C | 0.44 | -1.73E-01(0.006) | 1.24E-165 |
| 14 | rs17740413 | 54798528 | T/C | 0.09 | -1.50E-01(0.011) | 4.21E-39 |
| 14 | rs3825614 | 54799140 | A/G | 0.33 | -1.41E-01(0.007) | 7.05E-97 |
| 14 | rs10140801 | 54800156 | T/C | 0.42 | -1.76E-01(0.006) | 4.29E-173 |
| 14 | rs6573013 | 54805624 | G/T | 0.44 | -1.72E-01(0.006) | 8.43E-165 |
| 14 | rs6573014 | 54805644 | C/T | 0.44 | -1.73E-01(0.006) | 5.04E-166 |
| 14 | rs2341622 | 54806996 | A/C | 0.44 | -1.73E-01(0.006) | 5.09E-166 |
| 14 | rs2880103 | 54807584 | C/A | 0.34 | -1.38E-01(0.007) | 3.21E-94 |
| 14 | rs10146637 | 54814564 | A/G | 0.33 | -1.39E-01(0.007) | 3.21E-95 |
| 14 | rs10141552 | 54815844 | C/T | 0.43 | -1.75E-01(0.006) | 6.31E-167 |
| 14 | rs8021583 | 54819360 | A/G | 0.44 | -1.73E-01(0.006) | 5.60E-167 |
| 14 | rs11158034 | 54823312 | T/C | 0.33 | -1.39E-01(0.007) | 3.95E-95 |
| 14 | rs17672950 | 54825972 | A/G | 0.09 | -1.50E-01(0.011) | 4.76E-39 |
| 14 | rs11625001 | 54827912 | C/T | 0.44 | -1.73E-01(0.006) | 8.61E-167 |
| 14 | rs17740741 | 54832464 | C/G | 0.42 | -1.76E-01(0.006) | 2.58E-173 |
| 14 | rs7158768 | 54833364 | C/A | 0.43 | -1.72E-01(0.006) | 3.08E-165 |
| 14 | rs8023197 | 54837148 | T/G | 0.44 | -1.73E-01(0.006) | 7.68E-167 |
| 14 | rs7159824 | 54842288 | T/C | 0.34 | -1.38E-01(0.007) | 7.28E-95 |
| 14 | rs11628437 | 54845220 | A/G | 0.44 | -1.73E-01(0.006) | 1.20E-166 |
| 14 | rs10129505 | 54846128 | T/C | 0.34 | -1.39E-01(0.007) | 7.29E-95 |
| 14 | rs7147247 | 54847464 | T/A | 0.34 | -1.39E-01(0.007) | 6.44E-95 |
| 14 | rs4580079 | 54850120 | A/G | 0.34 | -1.39E-01(0.007) | 5.84E-95 |
| 14 | rs2341534 | 54854488 | G/C | 0.34 | 8.58E-02(0.008) | 2.10E-27 |
| 14 | rs1972221 | 54855136 | A/C | 0.34 | -1.39E-01(0.007) | 2.82E-95 |
| 14 | rs7151581 | 54858200 | T/C | 0.34 | -1.39E-01(0.007) | 3.18E-95 |
| 14 | rs2026633 | 54858928 | A/G | 0.33 | -1.39E-01(0.007) | 2.09E-95 |
| 14 | rs10134317 | 54863200 | T/C | 0.34 | -1.39E-01(0.007) | 2.59E-95 |
| 14 | rs10140869 | 54865036 | A/G | 0.42 | -1.77E-01(0.006) | 2.13E-175 |
| 14 | rs8005450 | 54868920 | T/A | 0.33 | -1.39E-01(0.007) | 2.26E-95 |
| 14 | rs9323280 | 54871440 | C/A | 0.10 | -1.41E-01(0.011) | 4.18E-38 |
| 14 | rs6573017 | 54871996 | A/G | 0.44 | -1.74E-01(0.006) | 1.37E-168 |
| 14 | rs17741542 | 54875616 | A/G | 0.44 | -1.74E-01(0.006) | 1.49E-168 |
| 14 | rs17741560 | 54875648 | T/C | 0.05 | -4.85E-01(0.044) | 7.67E-28 |
| 14 | rs7154889 | 54876640 | A/G | 0.44 | -1.74E-01(0.006) | 2.02E-168 |
| 14 | rs17673930 | 54877904 | G/A | 0.09 | -1.54E-01(0.011) | 1.89E-40 |
| 14 | rs17741681 | 54880468 | G/A | 0.09 | -1.55E-01(0.012) | 2.42E-40 |
| 14 | rs7152390 | 54881456 | T/C | 0.34 | -1.39E-01(0.007) | 1.77E-95 |
| 14 | rs8006525 | 54883148 | G/T | 0.44 | -1.75E-01(0.006) | 3.17E-168 |
| 14 | rs9919926 | 54883644 | C/G | 0.42 | -1.79E-01(0.006) | 1.41E-175 |
| 14 | rs9919932 | 54883832 | G/A | 0.34 | -1.40E-01(0.007) | 2.79E-95 |
| 14 | rs7148669 | 54884100 | G/A | 0.27 | 1.02E-01(0.007) | 4.01E-44 |
| 14 | rs10134339 | 54884132 | C/T | 0.44 | -1.75E-01(0.006) | 4.22E-168 |
| 14 | rs17741825 | 54884512 | T/G | 0.09 | -1.54E-01(0.011) | 2.25E-40 |
| 14 | rs11851169 | 54885684 | A/G | 0.44 | -1.74E-01(0.006) | 5.21E-168 |
| 14 | rs11851870 | 54885736 | C/A | 0.18 | -1.39E-01(0.008) | 3.40E-63 |
| 14 | rs17741831 | 54886468 | A/T | 0.44 | -1.76E-01(0.006) | 1.57E-168 |
| 14 | rs10141396 | 54886680 | G/T | 0.34 | -1.39E-01(0.007) | 9.49E-96 |
| 14 | rs10144418 | 54887460 | C/T | 0.42 | -1.78E-01(0.006) | 4.86E-176 |
| 14 | rs1045002 | 54888272 | A/T | 0.44 | -1.75E-01(0.006) | 3.99E-168 |
| 14 | rs3742569 | 54888460 | C/T | 0.44 | -1.74E-01(0.006) | 4.39E-168 |
| 14 | rs1045004 | 54889544 | A/G | 0.20 | -1.34E-01(0.008) | 3.52E-63 |
| 14 | rs7153110 | 54890380 | G/A | 0.44 | -1.74E-01(0.006) | 1.42E-168 |
| 14 | rs7159144 | 54891076 | A/G | 0.44 | -1.74E-01(0.006) | 2.23E-168 |
| 14 | rs10150760 | 54891300 | G/A | 0.44 | -1.74E-01(0.006) | 2.42E-168 |
| 14 | rs11621265 | 54891848 | T/G | 0.29 | -1.63E-01(0.007) | 6.72E-122 |
| 14 | rs7144737 | 54892700 | G/A | 0.44 | -1.74E-01(0.006) | 2.52E-168 |
| 14 | rs17674463 | 54894616 | T/A | 0.44 | -1.74E-01(0.006) | 3.76E-168 |
| 14 | rs7147136 | 54896440 | C/G | 0.42 | -1.77E-01(0.006) | 4.77E-174 |
| 14 | rs10147765 | 54897580 | A/G | 0.42 | -1.78E-01(0.006) | 1.64E-174 |
| 14 | rs7158791 | 54898360 | G/A | 0.34 | -1.40E-01(0.007) | 1.06E-95 |
| 14 | rs17674563 | 54900880 | A/G | 0.42 | -1.79E-01(0.006) | 8.33E-176 |
| 14 | rs2297816 | 54902792 | G/A | 0.34 | -1.41E-01(0.007) | 8.18E-97 |
| 14 | rs1572611 | 54903628 | A/T | 0.45 | -1.86E-01(0.006) | 7.36E-175 |
| 14 | rs10136596 | 54906528 | G/A | 0.44 | -1.75E-01(0.006) | 2.18E-168 |
| 14 | rs10142448 | 54907860 | C/A | 0.44 | -1.75E-01(0.006) | 3.69E-168 |
| 14 | rs2009291 | 54907984 | A/C | 0.44 | -1.75E-01(0.006) | 3.50E-168 |
| 14 | rs7150763 | 54909552 | G/A | 0.33 | -1.39E-01(0.007) | 3.12E-95 |
| 14 | rs10131730 | 54909844 | T/C | 0.34 | -1.39E-01(0.007) | 2.26E-95 |
| 14 | rs9323283 | 54910536 | T/C | 0.17 | -1.67E-01(0.011) | 8.58E-52 |
| 14 | rs8013713 | 54911004 | T/C | 0.44 | -1.74E-01(0.006) | 3.17E-168 |
| 14 | rs8015211 | 54911200 | C/T | 0.34 | -1.39E-01(0.007) | 1.73E-95 |
| 14 | rs8014477 | 54911232 | A/G | 0.34 | -1.39E-01(0.007) | 2.15E-95 |
| 14 | rs8014540 | 54911440 | A/C | 0.33 | -1.39E-01(0.007) | 1.90E-95 |
| 14 | rs10137307 | 54914936 | C/T | 0.42 | -1.78E-01(0.006) | 9.10E-171 |
| 14 | rs946059 | 54917220 | G/A | 0.44 | -1.74E-01(0.006) | 3.45E-168 |
| 14 | rs3783650 | 54918724 | C/A | 0.44 | -1.74E-01(0.006) | 1.69E-167 |
| 14 | rs3783651 | 54920728 | C/G | 0.33 | -1.39E-01(0.007) | 1.25E-95 |
| 14 | rs4597235 | 54921740 | G/A | 0.44 | -1.72E-01(0.006) | 2.95E-166 |
| 14 | rs3825616 | 54923040 | C/G | 0.43 | -1.74E-01(0.006) | 4.06E-168 |
| 14 | rs17742621 | 54923476 | C/T | 0.10 | -1.30E-01(0.011) | 1.56E-32 |
| 14 | rs3783652 | 54923960 | G/A | 0.43 | -1.75E-01(0.006) | 2.67E-169 |
| 14 | rs3783653 | 54924760 | A/G | 0.17 | -1.67E-01(0.011) | 8.53E-52 |
| 14 | rs17742719 | 54925348 | T/G | 0.18 | -1.40E-01(0.008) | 2.16E-61 |
| 14 | rs17675052 | 54925456 | T/C | 0.08 | -1.55E-01(0.012) | 4.16E-38 |
| 14 | rs9323285 | 54933584 | A/C | 0.34 | -1.38E-01(0.007) | 1.31E-93 |
| 14 | rs8003279 | 54933884 | G/A | 0.32 | -1.39E-01(0.007) | 5.40E-93 |
| 14 | rs6573020 | 54941204 | T/C | 0.43 | -1.73E-01(0.006) | 2.53E-167 |
| 14 | rs946057 | 54947152 | T/A | 0.39 | -1.61E-01(0.006) | 3.61E-132 |
| 14 | rs1538257 | 54949032 | G/T | 0.39 | -1.60E-01(0.006) | 7.54E-130 |
| 14 | rs10138446 | 54949440 | G/A | 0.38 | -1.63E-01(0.006) | 3.44E-130 |
| 14 | rs11624331 | 54949580 | G/C | 0.39 | -1.57E-01(0.006) | 8.78E-127 |
| 14 | rs1009647 | 54949800 | A/G | 0.27 | -1.27E-01(0.007) | 1.49E-70 |
| 14 | rs1009648 | 54949920 | C/T | 0.28 | -1.28E-01(0.007) | 2.52E-71 |
| 14 | rs1572610 | 54950096 | G/C | 0.29 | 9.65E-02(0.008) | 1.15E-32 |
| 14 | rs1890256 | 54951028 | T/C | 0.27 | -1.30E-01(0.007) | 2.18E-71 |
| 14 | rs8010013 | 54954580 | A/C | 0.55 | -8.55E-02(0.007) | 1.10E-38 |
| 14 | rs8014526 | 54954808 | C/G | 0.54 | -8.53E-02(0.007) | 1.82E-38 |
| 14 | rs8003684 | 54961792 | C/A | 0.22 | -1.25E-01(0.009) | 4.22E-41 |
| 14 | rs8016239 | 54964336 | C/A | 0.09 | -6.34E-02(0.012) | 4.20E-08 |
| 14 | rs17743484 | 54967252 | C/T | 0.13 | -1.54E-01(0.013) | 9.62E-34 |
| 14 | rs4470077 | 54967292 | G/A | 0.22 | -1.25E-01(0.009) | 6.13E-41 |
| 14 | rs10146736 | 54967676 | T/C | 0.19 | -1.42E-01(0.01) | 4.25E-43 |
| 14 | rs11848576 | 54971036 | T/C | 0.06 | -9.75E-02(0.017) | 2.21E-08 |
| 14 | rs1959443 | 54971072 | G/A | 0.13 | -1.53E-01(0.013) | 1.31E-33 |
| 14 | rs4901562 | 54971128 | G/A | 0.55 | -8.45E-02(0.007) | 6.35E-37 |
| 14 | rs10483641 | 54971720 | A/T | 0.13 | -1.53E-01(0.013) | 3.79E-33 |
| 14 | rs7149317 | 54972296 | G/A | 0.39 | -4.60E-02(0.007) | 5.20E-11 |
| 14 | rs1953739 | 54973608 | C/T | 0.07 | -8.48E-02(0.015) | 3.00E-08 |
| 14 | rs713475 | 54974404 | G/T | 0.13 | -1.53E-01(0.013) | 3.45E-33 |
| 14 | rs713473 | 54974448 | T/C | 0.13 | -1.53E-01(0.013) | 3.20E-33 |
| 14 | rs8014621 | 54976600 | A/C | 0.54 | -8.27E-02(0.006) | 3.82E-37 |
| 14 | rs8019270 | 54976924 | C/G | 0.44 | -4.96E-02(0.007) | 3.04E-14 |
| 14 | rs7142204 | 54978188 | A/C | 0.54 | -8.25E-02(0.006) | 9.89E-37 |
| 14 | rs1572613 | 54979648 | C/T | 0.54 | -8.25E-02(0.007) | 9.97E-37 |
| 14 | rs4901568 | 54982544 | T/A | 0.54 | -8.23E-02(0.006) | 1.36E-36 |
| 14 | rs4901569 | 54982632 | C/T | 0.45 | -4.57E-02(0.007) | 3.56E-12 |
| 14 | rs8015252 | 54985144 | A/G | 0.13 | -1.50E-01(0.013) | 1.13E-32 |
| 14 | rs17128488 | 54987288 | C/G | 0.13 | -1.51E-01(0.013) | 1.06E-32 |
| 14 | rs7143800 | 54987488 | A/G | 0.46 | -4.32E-02(0.007) | 3.51E-11 |
| 14 | rs998871 | 54988056 | G/A | 0.46 | -4.08E-02(0.007) | 3.20E-10 |
| 14 | rs7146055 | 55035476 | A/G | 0.44 | -4.23E-02(0.007) | 2.93E-10 |
| 14 | rs1307315 | 55037548 | C/T | 0.32 | -5.80E-02(0.007) | 6.74E-16 |
| 14 | rs1188074 | 55041584 | T/C | 0.43 | -4.11E-02(0.007) | 6.18E-10 |
| 14 | rs1188076 | 55044228 | G/A | 0.32 | -5.60E-02(0.007) | 2.54E-15 |
| 14 | rs17745017 | 55048576 | A/G | 0.11 | -6.32E-02(0.011) | 6.75E-09 |
| 14 | rs1009714 | 55049292 | A/T | 0.43 | -4.11E-02(0.007) | 8.00E-10 |
| 14 | rs10134019 | 55066744 | T/G | 0.46 | -3.68E-02(0.007) | 7.86E-08 |
| 14 | rs8011732 | 55075920 | A/G | 0.43 | -3.80E-02(0.007) | 1.62E-08 |
| 14 | rs942317 | 55093576 | A/C | 0.44 | -3.80E-02(0.007) | 1.50E-08 |
| 14 | rs6573053 | 55223060 | T/C | 0.38 | -3.70E-02(0.007) | 6.74E-08 |
| 14 | rs7147303 | 55245388 | T/C | 0.23 | -4.88E-02(0.008) | 1.31E-09 |
| 14 | rs2342585 | 55246740 | A/T | 0.23 | -4.87E-02(0.008) | 1.35E-09 |
| 14 | rs2342586 | 55246840 | A/G | 0.23 | -4.85E-02(0.008) | 1.60E-09 |
| 14 | rs8021103 | 55247116 | T/G | 0.23 | -4.80E-02(0.008) | 2.18E-09 |
| 14 | rs10498477 | 55249852 | A/G | 0.22 | -4.76E-02(0.008) | 1.95E-09 |
| 14 | rs10130783 | 55251272 | A/G | 0.23 | -4.85E-02(0.008) | 1.36E-09 |
| 14 | rs11158050 | 55275968 | T/A | 0.21 | -6.72E-02(0.01) | 1.59E-11 |
| 14 | rs945269 | 55276464 | G/A | 0.48 | -1.08E-01(0.015) | 2.48E-12 |
| 14 | rs7148653 | 55430040 | A/G | 0.07 | -8.00E-02(0.015) | 5.36E-08 |
| 14 | rs17128903 | 55438128 | A/C | 0.07 | -8.00E-02(0.013) | 6.99E-10 |
| 14 | rs17128904 | 55438816 | G/C | 0.06 | -8.38E-02(0.014) | 2.63E-09 |
| 14 | rs1112305 | 55446000 | T/C | 0.07 | -8.91E-02(0.016) | 3.25E-08 |

BP: base pair; AF: Allele Frequency; Effect (se): beta coefficient of individual SNP on plasma galectin-3 levels; (se): standard error.
