# Supplementary material for: A Genome-Wide Association Study of Circulating Galectin-3
Source: PLoS One. 2012 Oct 9;7(10):e47385. doi: 10.1371/journal.pone.0047385 (PMC3467202; doi:10.1371/journal.pone.0047385)
Supplement: Table S2 — Previous reported traits with genome wide associations at the ABO locus. (DOC) [file pone.0047385.s002.doc]

**Table S2.** Previous reported traits with genome wide associations at the ABO locus.

| **Disease/Trait** | **Position on Chr 9** | **Mapped gene** | **Strongest SNP** | **Risk Allele** | **R2 with rs644234** | **Context** | **Risk Allele Frequency** | **P-value** | **OR or beta** | **95% CI** | **First Author** |
| --- | --- | --- | --- | --- | --- | --- | --- | --- | --- | --- | --- |
| mean corpuscular hemoglobin concentration (MCHC) | 136131322 | ABO | rs8176746 | T | 0.1 | cds-synon | 0.18 | 4.00E-08 | 0.21 | [NR] % variance | Kamatani |
| D-dimer | 136137065 | ABO | rs687621 | G | 0.9 | intron | 0.317 | 7.00E-06 | 0.03 | [NR] % increase | Smith |
| Campesterol | 136139265 | ABO | rs657152 | T | 1.0 | intron | 0.383 | 9.00E-13 | 8 | [NR] % increase | Teupser |
| Alkaline phosphatase | 136139265 | ABO | rs657152 | T | 1.0 | intron | 0.39 | 2.00E-30 | 0.05 | [0.039-0.055] U/L decrease | Yuan |
| Coronary heart disease | 136142203 | ABO | rs514659 | C | 0.9 | intron | 0.37 | 8.00E-09 | 1.21 | [1.13-1.28] | Reilly |
| IL-6 | 136142355 | ABO | rs643434 | A | 1.0 | intron | 0.258 | 9.00E-25 | NR | NR | Naitza |
| ADpSGEGDFXAEGGGVR/ADSGEGDFXAEGGGVR | 136143442 | ABO | rs612169 | G | 0.9 | intron | 0.335 | 9.00E-40 | 0.2 | [NR] unit increase | Suhre |
| Duodenal ulcer | 136149229 | ABO | rs505922 | T | 0.9 | intron | 0.54 | 1.00E-10 | 1.32 | [NR] | Tanikawa |
| Venous thromboembolism | 136149229 | ABO | rs505922 | C | 0.9 | intron | 0.43 | 1.00E-34 | 1.92 | [NR] | Germain |
| Graves' disease | 136149229 | ABO | rs505922 | T | 0.9 | intron | 0.53 | 8.00E-06 | 1.13 | [1.07-1.20] | Chu |
| Pancreatic cancer | 136149229 | ABO | rs505922 | C | 0.9 | intron | 0.35 | 5.00E-08 | 1.2 | [1.12-1.28] | Amundadottir |
| Venous thromboembolism | 136149229 | ABO | rs505922 | C | 0.9 | intron | 0.35 | 4.00E-15 | 1.81 | [1.56-2.11] | Tregouet |
| TNF-alpha | 136149229 | ABO | rs505922 | ? | 0.9 | intron | 0.34 | 7.00E-40 | NR | NR | Melzer |
| ICAM-1 | 136149399 | ABO | rs507666 | A | NA | intron | 0.2 | 3.00E-91 | 17.3 | [NR] ng/mL decrease | Pare |
| ICAM-1 | 136149399 | ABO | rs507666 | G | NA | intron | 0.2 | 5.00E-29 | 17.73 | [NR] umol/L decrease | Pare |
| LDL cholesterol | 136153875 | ABO - LCN1L2 | rs651007 | A | 0.4 | Intergenic | 0.26 | 6.00E-09 | 2.28 | [1.51-3.05] mg/dL increase | Kim |
| E-selectin | 136153875 | ABO - LCN1L2 | rs651007 | T | 0.4 | Intergenic | 0.22 | 2.00E-82 | 9.71 | [NR] % variance explained | Qi |
| Alkaline phosphatase | 136154168 | ABO - LCN1L2 | rs579459 | T | 0.4 | Intergenic | 0.8 | 3.00E-123 | 8.8 | [7.40-10.2] % increase | Chambers |
| Coronary heart disease | 136154168 | ABO - LCN1L2 | rs579459 | C | 0.4 | Intergenic | 0.21 | 4.00E-14 | 1.1 | [1.07-1.13] | Schunkert |
| P-Selectin | 136154168 | ABO - LCN1L2 | rs579459 | T | 0.4 | Intergenic | NR | 2.00E-41 | 14 | [12.04-15.96] % increase | Barbalic |
| E-selectin | 136154168 | ABO - LCN1L2 | rs579459 | C | 0.4 | Intergenic | 0.2 | 1.00E-29 | NR | NR | Paterson |
| ICAM-1 | 136154304 | ABO - LCN1L2 | rs649129 | T | 0.4 | Intergenic | NR | 1.00E-15 | 3.95 | [2.99-4.91] % decrease | Barbalic |
| Hematocrit (Ht) | 136154867 | ABO - LCN1L2 | rs495828 | T | 0.4 | Intergenic | 0.28 | 6.00E-10 | 0.26 | [NR] % variance | Kamatani |
| Hemoglobin (Hgb) | 136154867 | ABO - LCN1L2 | rs495828 | T | 0.4 | Intergenic | 0.28 | 1.00E-11 | 0.32 | [NR] % variance | Kamatani |
| Red blood cells (RBC) | 136154867 | ABO - LCN1L2 | rs495828 | T | 0.4 | Intergenic | 0.28 | 3.00E-12 | 0.33 | [NR] % variance | Kamatani |
| Alkaline phosphatase | 136154867 | ABO - LCN1L2 | rs495828 | T | 0.4 | Intergenic | 0.28 | 4.00E-59 | 3.79 | [NR] % variance | Kamatani |
| Angiotensin-converting enzyme activity | 136154867 | ABO - LCN1L2 | rs495828 | A | 0.4 | Intergenic | 0.17 | 3.00E-08 | 4.9 | [NR] % variance | Chung |
| Total cholesterol | 136155000 | ABO - LCN1L2 | rs635634 | T | 1.0 | Intergenic | 0.21 | 9.00E-21 | 2.3 | [1.81-2.79] mg/dL increase | Teslovich |
| LDL cholesterol | 136155000 | ABO - LCN1L2 | rs635634 | T | 1.0 | Intergenic | 0.22 | 8.00E-22 | 2.05 | [1.64-2.46] mg/dL increase | Teslovich |

**References Table S2**

1. Kamatani Y, Matsuda K, Okada Y, Kubo M, Hosono N, et al. (2010) Genome-wide association study of hematological and biochemical traits in a Japanese population. Nat Genet 42: 210-215.

2. Smith NL, Huffman JE, Strachan DP, Huang J, Dehghan A, et al. (2011) Genetic predictors of fibrin D-dimer levels in healthy adults. Circulation 123: 1864-1872.

3. Teupser D, Baber R, Ceglarek U, Scholz M, Illig T, et al. (2010) Genetic regulation of serum phytosterol levels and risk of coronary artery disease. Circ Cardiovasc Genet 3: 331-339.

4. Yuan X, Waterworth D, Perry JR, Lim N, Song K, et al. (2008) Population-based genome-wide association studies reveal six loci influencing plasma levels of liver enzymes. Am J Hum Genet 83: 520-528.

5. Reilly MP, Li M, He J, Ferguson JF, Stylianou IM, et al. (2011) Identification of ADAMTS7 as a novel locus for coronary atherosclerosis and association of ABO with myocardial infarction in the presence of coronary atherosclerosis: two genome-wide association studies. Lancet 377: 383-392.

6. Naitza S, Porcu E, Steri M, Taub DD, Mulas A, et al. (2012) A genome-wide association scan on the levels of markers of inflammation in Sardinians reveals associations that underpin its complex regulation. PLoS Genet 8: e1002480.

7. Suhre K, Shin SY, Petersen AK, Mohney RP, Meredith D, et al. (2011) Human metabolic individuality in biomedical and pharmaceutical research. Nature 477: 54-60.

8. Tanikawa C, Urabe Y, Matsuo K, Kubo M, Takahashi A, et al. (2012) A genome-wide association study identifies two susceptibility loci for duodenal ulcer in the Japanese population. Nat Genet 44: 430-434, S431-432.

9. Germain M, Saut N, Greliche N, Dina C, Lambert JC, et al. (2011) Genetics of venous thrombosis: insights from a new genome wide association study. PLoS One 6: e25581.

10. Chu X, Pan CM, Zhao SX, Liang J, Gao GQ, et al. (2011) A genome-wide association study identifies two new risk loci for Graves' disease. Nat Genet 43: 897-901.

11. Amundadottir L, Kraft P, Stolzenberg-Solomon RZ, Fuchs CS, Petersen GM, et al. (2009) Genome-wide association study identifies variants in the ABO locus associated with susceptibility to pancreatic cancer. Nat Genet 41: 986-990.

12. Tregouet DA, Heath S, Saut N, Biron-Andreani C, Schved JF, et al. (2009) Common susceptibility alleles are unlikely to contribute as strongly as the FV and ABO loci to VTE risk: results from a GWAS approach. Blood 113: 5298-5303.

13. Melzer D, Perry JR, Hernandez D, Corsi AM, Stevens K, et al. (2008) A genome-wide association study identifies protein quantitative trait loci (pQTLs). PLoS Genet 4: e1000072.

14. Pare G, Ridker PM, Rose L, Barbalic M, Dupuis J, et al. (2011) Genome-wide association analysis of soluble ICAM-1 concentration reveals novel associations at the NFKBIK, PNPLA3, RELA, and SH2B3 loci. PLoS Genet 7: e1001374.

15. Pare G, Chasman DI, Kellogg M, Zee RY, Rifai N, et al. (2008) Novel association of ABO histo-blood group antigen with soluble ICAM-1: results of a genome-wide association study of 6,578 women. PLoS Genet 4: e1000118.

16. Kim YJ, Go MJ, Hu C, Hong CB, Kim YK, et al. (2011) Large-scale genome-wide association studies in East Asians identify new genetic loci influencing metabolic traits. Nat Genet 43: 990-995.

17. Qi L, Cornelis MC, Kraft P, Jensen M, van Dam RM, et al. (2010) Genetic variants in ABO blood group region, plasma soluble E-selectin levels and risk of type 2 diabetes. Hum Mol Genet 19: 1856-1862.

18. Chambers JC, Zhang W, Sehmi J, Li X, Wass MN, et al. (2011) Genome-wide association study identifies loci influencing concentrations of liver enzymes in plasma. Nat Genet 43: 1131-1138.

19. Schunkert H, Konig IR, Kathiresan S, Reilly MP, Assimes TL, et al. (2011) Large-scale association analysis identifies 13 new susceptibility loci for coronary artery disease. Nat Genet 43: 333-338.

20. Barbalic M, Dupuis J, Dehghan A, Bis JC, Hoogeveen RC, et al. (2010) Large-scale genomic studies reveal central role of ABO in sP-selectin and sICAM-1 levels. Hum Mol Genet 19: 1863-1872.

21. Paterson AD, Lopes-Virella MF, Waggott D, Boright AP, Hosseini SM, et al. (2009) Genome-wide association identifies the ABO blood group as a major locus associated with serum levels of soluble E-selectin. Arterioscler Thromb Vasc Biol 29: 1958-1967.

22. Chung CM, Wang RY, Chen JW, Fann CS, Leu HB, et al. (2010) A genome-wide association study identifies new loci for ACE activity: potential implications for response to ACE inhibitor. Pharmacogenomics J 10: 537-544.

23. Teslovich TM, Musunuru K, Smith AV, Edmondson AC, Stylianou IM, et al. (2010) Biological, clinical and population relevance of 95 loci for blood lipids. Nature 466: 707-713.
